# Integrating multi-omics and machine learning to uncover CCL20 as a potential regulator of the immunosuppressive microenvironment in lung adenocarcinoma

**DOI:** 10.3389/fimmu.2026.1870538

**Published:** 2026-07-01

**Authors:** Guishi Wang, Guichao Wang, Guanghui Xu, Zhenjiang Wu, Guolei Wang, Yanjun Dong

**Affiliations:** 1 Henan Provincial Chest Hospital, Zhengzhou, China; 2 Henan Medical University, Xinxiang, China; 3 First Affiliated Hospital of Henan University, Kaifeng, China

**Keywords:** CCL20, lung adenocarcinoma, multi-omics data, progression, tumor microenvironment

## Abstract

**Background:**

The interplay between malignant cells and the immunosuppressive tumor microenvironment (TME) is pivotal for lung adenocarcinoma (LUAD) progression.

**Methods:**

This study employed single-cell RNA sequencing, spatial transcriptomics, multi-omics analysis, and ensemble machine learning, combined with in vitro and in vivo functional experiments, to investigate the role of CCL20 in the immunosuppressive TME of LUAD.

**Results:**

We identified a distinct, metastasis-enriched malignant epithelial subpopulation characterized by a pro-inflammatory signature and high CCL20 expression. Pseudotime and regulon analyses suggested enrichment of NF-κB/STAT signaling regulon activity during malignant evolution. Spatial transcriptomics and cellular communication inference demonstrated that CCL20-high tumor cells co-localized with and actively recruited regulatory T cells (Tregs) via the specific CCL20-CCR6 ligand-receptor pair. Multi-omics analysis confirmed that high CCL20 expression correlated with increased Treg infiltration and served as an independent prognostic biomarker. An ensemble machine learning model based on the CCL20-CCR6 axis effectively stratified high-risk patients across multiple validation datasets. Functionally, genetic ablation of CCL20 attenuated the proliferative, migratory, and invasive capacities of LUAD cells *in vitro* and suppressed tumor growth *in vivo*.

**Conclusions:**

The primary causal evidence chain of this study centers on the functional validation of the CCL20-CCR6 axis in Treg chemotaxis; the upstream computational inference of NF-κB/STAT signaling regulation should be regarded as a hypothesis-generating exploration requiring further validation. CCL20 thus represents a potential prognostic biomarker and therapeutic target for LUAD.

## 1 Introduction

Lung adenocarcinoma (LUAD), the most prevalent histological subtype of non-small cell lung cancer, remains a leading cause of cancer-related mortality worldwide (1–4). Despite advances in targeted therapies and immunotherapy, a significant proportion of patients exhibit primary or acquired resistance, underscoring the urgent need to decipher the complex mechanisms underlying tumor progression and immune evasion (5–7). The tumor microenvironment (TME) is a dynamic ecosystem where malignant cells interact with diverse stromal and immune components, critically influencing therapeutic response and clinical outcome (8–11). Within this milieu, regulatory T cells (Tregs) are pivotal suppressors of anti-tumor immunity, and their infiltration is frequently associated with poor prognosis across various cancers, including LUAD (12, 13). However, the precise molecular mechanisms orchestrating the recruitment and spatial organization of Tregs within the LUAD TME are not fully elucidated.

Chemokines and their receptors constitute a fundamental signaling network that directs immune cell trafficking and positioning. The CCL20-CCR6 axis is a key pathway known to mediate the homing of CCR6-expressing lymphocytes, including Tregs, to sites of inflammation and cancer (14–17). While aberrant CCL20 expression has been implicated in the pathogenesis of several malignancies, its functional role, upstream regulatory drivers, and spatial dynamics within the LUAD ecosystem are poorly understood. Furthermore, it remains unclear whether CCL20 serves merely as a passive inflammatory marker or acts as an active orchestrator connecting tumor-intrinsic malignant programs with the establishment of an immunosuppressive niche.

Recent technological advances, particularly single-cell and spatial transcriptomics, offer unprecedented resolution to dissect cellular heterogeneity, trace cell-state transitions, and map intercellular communication within intact tissues (18–22). Integrating these high-resolution tools with multi-omics data from bulk cohorts and functional validation presents a powerful strategy to bridge the gap between descriptive association and mechanistic insight.

In this study, we hypothesize that a specific malignant epithelial subpopulation, emerging during LUAD progression, drives immunosuppression by secreting CCL20 to recruit Tregs. We employed an integrative framework combining single-cell RNA sequencing (scRNA-seq), spatial transcriptomics, multi-omics correlation analysis, and machine learning to systematically investigate this axis. We further validated its functional significance through *in vitro* and *in vivo* models. Our work aims to delineate a coherent pathway from tumor cell evolution to immune microenvironment remodeling, positioning CCL20 not only as a mechanistic linchpin but also as a potential prognostic biomarker and therapeutic target in LUAD.

## 2 Methods

### 2.1 Data acquisition and preprocessing

The RNA sequencing transcriptomic profiles and associated clinical metadata for LUAD were acquired from The Cancer Genome Atlas (TCGA) database through the GDC data portal. Gene expression values were converted to Transcripts Per Million (TPM) units to standardize downstream analyses. For independent validation, multiple microarray datasets (GSE75037, GSE10072, GSE72094, GSE118370) were obtained from the Gene Expression Omnibus (GEO) repository. Batch effects arising from platform differences were mitigated using the “ComBat” algorithm implemented in the sva R package. To investigate heterogeneity within the TME, publicly accessible scRNA-seq and spatial transcriptomics datasets (GSE307534, GSE189487, GSE153935) were additionally utilized.

### 2.2 Weighted gene co-expression network analysis

To identify gene modules exhibiting significant associations with clinical traits, including Treg cell infiltration scores and survival status, a WGCNA was performed using the corresponding R package. Initial data preprocessing involved the removal of outlier samples through hierarchical clustering. An optimal soft-thresholding power (β) was selected to approximate a scale-free network topology. The adjacency matrix, derived from pairwise gene correlations, was subsequently converted into a Topological Overlap Matrix (TOM) to quantify network interconnectedness. Gene modules were delineated using a dynamic tree-cutting algorithm with a minimum module size of 30 genes. The module eigengenes (MEs), representing the first principal component of each module, were correlated with clinical parameters to pinpoint the most relevant module. Within this key module, genes demonstrating a module membership (MM) > 0.8 and gene significance (GS) > 0.2 for the trait of interest were designated as hub genes.

### 2.3 Identification of robust biomarkers via integrated machine learning

To systematically identify robust and reproducible diagnostic and prognostic biomarkers, we integrated three distinct machine learning algorithms. The Least Absolute Shrinkage and Selection Operator (LASSO) regression was employed using the glmnet package with 10-fold cross-validation to penalize coefficients and mitigate overfitting. Subsequently, a Support Vector Machine-Recursive Feature Elimination (SVM-RFE) analysis was implemented via the e1071 package to rank genes according to their contribution weights. Furthermore, a Random Forest (RF) algorithm was applied using the randomForest package, where feature importance was assessed based on the Mean Decrease Gini index. Genes consistently identified as candidate features by all three algorithms were selected for subsequent validation.

### 2.4 Evaluation of immune cell infiltration

To assess the relative enrichment of 28 distinct immune cell populations within the TME, we employed the Single-Sample Gene Set Enrichment Analysis (ssGSEA) algorithm implemented in the GSVA R package. The gene signatures defining these immune cell types were sourced from the TISIDB database. Subsequently, Spearman’s rank correlation analysis was conducted to evaluate the associations between individual gene expression levels and the corresponding immune cell infiltration scores.

### 2.5 scRNA-seq analysis

scRNA-seq data were processed and analyzed using the Seurat R package. Quality control was performed by filtering out cells with an insufficient number of detected genes or an excessively high percentage of mitochondrial gene counts. Expression data were normalized using the LogNormalize method. The top 2,000 highly variable genes were identified and subjected to principal component analysis (PCA). For dimensionality reduction and visualization, Uniform Manifold Approximation and Projection (UMAP) was employed. Cell clusters were annotated based on the expression of canonical marker genes.

To discriminate malignant from non-malignant cells, chromosomal copy number variations (CNVs) were inferred using the infer CNV package, utilizing immune cells as a reference.

The pseudotemporal trajectory of tumor cells was constructed using the Monocle 2 package. Differential expression analysis along the pseudotime axis was performed to identify genes associated with the tumor evolutionary trajectory.

To reconstruct gene regulatory networks, we applied the SCENIC workflow (Python implementation). The activity of regulons (transcription factors and their target gene sets) was quantified using the AUCell scoring method.

### 2.6 Cell-cell communication analysis

To decipher intercellular communication networks within the TME, we employed the Cell Chat R package. Normalized scRNA-seq data were utilized as input, and gene expression was projected onto a curated database of ligand-receptor interactions. Communication probabilities were calculated to identify statistically significant ligand-receptor interactions (CCL20-CCR6) between distinct tumor subpopulations and T cells. The inferred interaction networks were visualized using circle plots and bubble plots to depict both the strength of cellular crosstalk.

### 2.7 Spatial transcriptomics analysis

To validate the spatial co-localization of specific cell types, spatial transcriptomics data were processed and analyzed using Seurat. Gene signatures derived from scRNA-seq analyses, such as the Tumor_Inflammatory score and Treg cell score, were integrated with the spatial data by projecting them via the Add Module Score function. The spatial distributions of these signature scores were then overlaid onto the corresponding histological sections for visualization. To statistically quantify cellular proximity, we computed the spatial distances between spots enriched for distinct signatures.

### 2.8 Construction and validation of prognostic models

To construct a robust consensus prognostic model, we implemented an integrative machine learning framework. Multiple algorithmic combinations—including Random Survival Forests (RSF), Lasso, Ridge, Elastic-Net (Enet), Stepwise Cox (StepCox), CoxBoost, Partial Least Squares Regression (PLS-R), Gradient Boosting Machine (GBM), and Support Vector Machine (SVM)—were systematically evaluated across the training and validation cohorts. The concordance index (C-index) was computed for each candidate model to assess predictive performance. The ensemble model demonstrating the highest average C-index (an integration of Random Forest and GBM) was selected as the final prognostic classifier. Subsequently, a continuous risk score was calculated for each patient. Kaplan-Meier survival analysis was then performed to compare overall survival between the defined high- and low-risk groups.

### 2.9 Cell line culturing

the human LUAD cell lines A549 and H1299 were obtained from Pricella Biotechnology (Wuhan, China). Cultures were maintained in RPMI-1640 medium (Gibco, USA) containing 10% fetal bovine serum (FBS; Gibco) and 1% penicillin/streptomycin (Gibco), and were incubated at 37 °C in a humidified atmosphere of 5% CO_2_. Cell line authentication was performed using short tandem repeat (STR) profiling.

### 2.10 Protein expression analysis via western blot

Western blot analysis was performed following established protocols. The primary antibodies employed in this study were as follows: a rabbit monoclonal antibody targeting CCL20 (CST, # 76924) used at a 1:1000 dilution, and a mouse monoclonal anti-β-actin antibody (Proteintech, 66009-1-Ig) applied at 1:10,000. For detection, horseradish peroxidase (HRP)-conjugated secondary antibodies from goat were utilized: anti-mouse (Proteintech, RGAM001; 1:10,000) and anti-rabbit (Proteintech, RGAR001; 1:10,000).

### 2.11 Establishment of stably transfected cell lines

Lentiviral vectors expressing short hairpin RNAs targeting CCL20 (sh-CCL20) and a non-targeting control (shNC) were purchased from Genechem Co., Ltd (Shanghai, China). The specific shRNA sequences were: sh-CCL20#1: AAAA GAGTTTGCTCCTGGCTGCTTTGATGTTGGATCCAACATCAAAGCAGCCAGGAGCAAACTC; sh-CCL20#2: AAAAGCAACTTTGACTGCTGTCTTGGATATTGGATCCAATATCCAAGACAGCAGTCAAAGTTGC. A549 and H1299 cell lines were seeded into 6-well plates and transduced using lentiviral vectors when reaching approximately 20–30% confluency. After a 72-hour incubation period, cells stably integrating the constructs were selected with puromycin (1 μg/mL, Beyotime, #ST551) for one week. Verification of successful gene knockdown was subsequently performed via Western blot analysis in both cellular models.

### 2.12 Evaluation of cellular proliferation

To assess cellular proliferation, we employed a Cell Counting Kit-8 (CCK-8) assay. Stable A549 and H1299 cells (1.5 × 10³ cells per well) were plated in 96-well plates. Cellular viability was quantified at the indicated time points (0, 24, 48, 72, and 96 hours) by incubating the cells with 10 μl of CCK-8 reagent (Abbkine, #BMU106-CN) per well for 2 hours under light-protected conditions. All measurements were conducted in triplicate.

Colony formation capacity was evaluated by seeding cells in 6-well plates at a density of 1 × 10³ cells per well. Following a 14-day culture period, during which the medium was refreshed every 7 days, the resulting colonies were fixed with 4% paraformaldehyde (Biosharp, #BL539A) and stained with 0.5% crystal violet (Phygene, #PH1322). Colonies were subsequently imaged and counted. This experiment was independently performed in three biological replicates.

### 2.13 Analysis of migration and invasion

To assess cellular migratory capacity, wound healing assays were performed. A549 and H1299 cells were cultured in 6-well plates until forming a confluent monolayer. Following a 12-hour serum starvation period, a uniform scratch wound was generated using a sterile 200 μl pipette tip. After washing, cells were incubated in medium containing 2% FBS. Images of the wound area were documented at 0 and 24 hours post-scratching, and wound closure was measured using ImageJ software. All experiments were conducted with three technical replicates.

Cell migration and invasive potential were further evaluated using a Transwell chamber system (CORNING, #3422). For the migration assay, cells were seeded in serum-free medium into the upper chamber of uncoated inserts. The invasion assay was performed using inserts pre-coated with Matrigel (BD Biosciences). Specifically, 4×10^4^ A549 cells or 5×10^4^ H1299 cells, suspended in serum-free medium, were added to the upper compartment. The lower chamber was filled with medium supplemented with 20% FBS as a chemoattractant. After incubation (24 hours for migration; 48 hours for invasion), cells that had traversed the membrane were fixed, stained with crystal violet, and quantified using an inverted light microscope (Leica). Each assay was independently repeated three times.

### 2.14 *In Vivo* subcutaneous tumor model

Animal studies were approved by the Medical Research Ethics Sub-committee of Henan University students(HUSOM2026-565). A cohort of five 4-week-old female nude mice (BALB/c-nu, Gem Pharmatech Co., Ltd) was maintained under specific pathogen-free conditions. For the tumorigenicity assay, each animal underwent bilateral subcutaneous injection in the axillary region with 5×10^6^ A549 cells transduced with either sh-CCL20#1 or a non-targeting shRNA control (sh-NC). Beginning on day 7 post-inoculation, tumor dimensions were recorded every other day, and volume was determined using the formula V = (length × width²)/2. At the experimental endpoint (day 29), euthanasia was performed by gradual displacement of chamber air with compressed CO_2_ at a flow rate set to 30-40% of the chamber volume per minute. Death was confirmed by cervical dislocation. Subsequently, tumors were excised and their wet weights were recorded.

### 2.15 Statistical methods

All statistical analyses and graphical representations were performed using R software (version 4.2.1) and the XianTao online platform (https://www.xiantao.love/). For comparisons involving continuous variables between two groups, data distribution was first assessed: normality was tested using the Shapiro-Wilk test, and homogeneity of variances was evaluated using Levene’s test. Parametric tests (Student’s t-test) were applied when both assumptions were met; otherwise, non-parametric alternatives (Wilcoxon rank-sum test) were employed. Categorical variables were compared using the Chi-square test or Fisher’s exact test, as appropriate. Correlation analyses were conducted using Pearson’s or Spearman’s coefficients based on data distribution. Survival differences were assessed via the log-rank test. Univariate and multivariate Cox proportional hazards regression analyses were utilized to identify independent prognostic factors. A two-sided p-value of less than 0.05 was considered statistically significant. The specific statistical test used for each analysis is indicated in the corresponding figure legend.

## 3 Results

### 3.1 scRNA-seq reveals TME heterogeneity and dynamic evolution of malignant epithelial subpopulations

**Figure 1:**
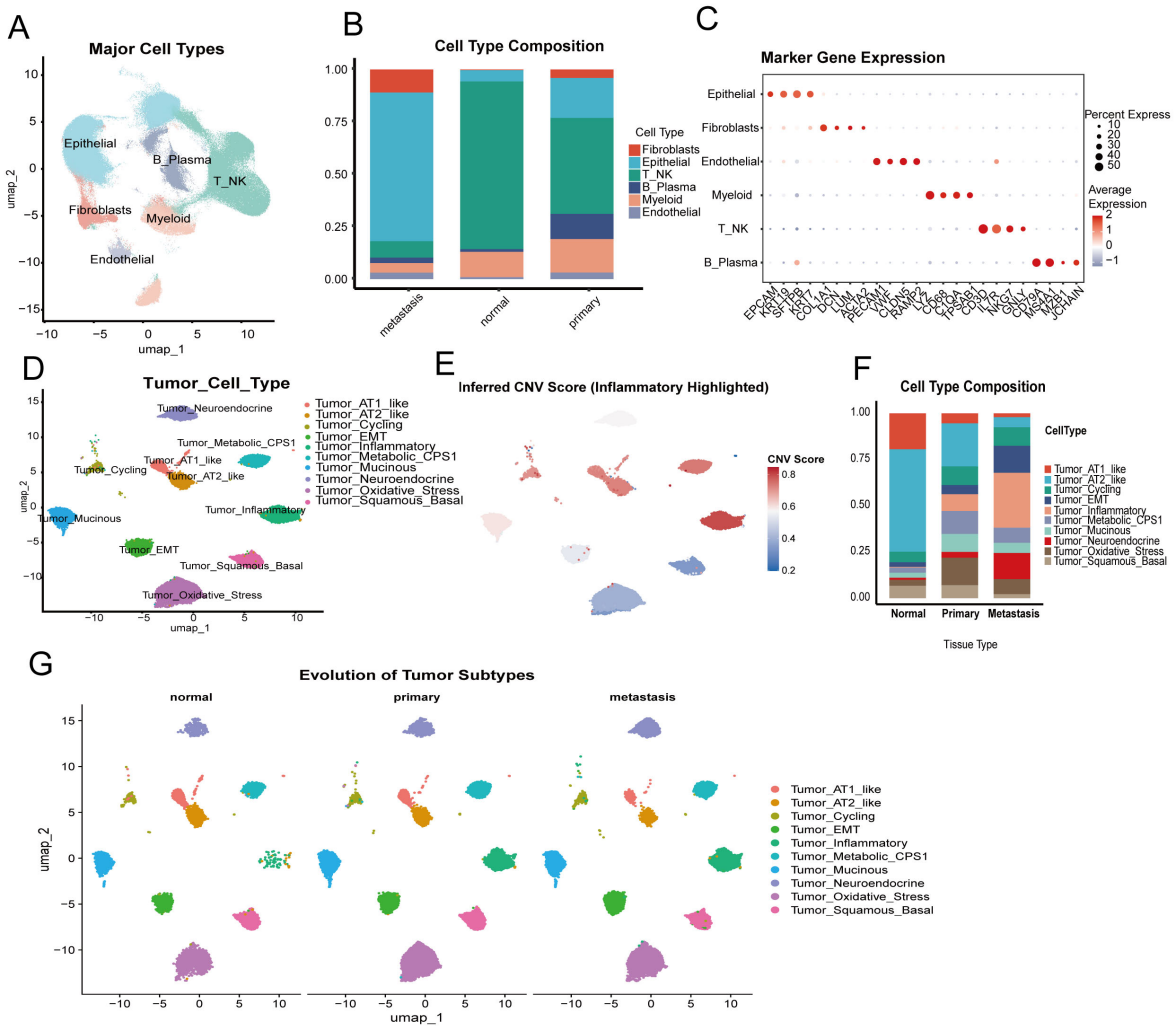
Single-cell atlas and tumor heterogeneity in LUAD. **(A)** UMAP visualization of major cell types, including epithelial cells, fibroblasts, myeloid cells, and T/NK cells. **(B)** Stacked bar plot depicting the proportional composition of major cell types across metastatic, normal adjacent, and primary tumor samples. **(C)** Bubble plot showing the expression of canonical marker genes used for cell type annotation. Dot size represents the fraction of expressing cells, and color intensity indicates the average expression level. **(D)** UMAP plot of tumor epithelial subclusters, identifying functionally distinct subpopulations such as Cycling, EMT, and Inflammatory tumor cells. **(E)** UMAP plot of inferred copy number variation (CNV) scores. Red indicates high CNV scores, highlighting subpopulations with malignant genomic features. **(F)** Bar graph quantifying the relative abundance of each tumor epithelial subpopulation across different tissue origins. **(G)** Grouped UMAP plots illustrating the distribution dynamics of tumor subpopulations along disease progression (from normal to primary to metastatic tissue).

To dissect the cellular composition and heterogeneity of the TME, we performed dimensionality reduction and clustering analysis on the scRNA-seq data. Initial annotation based on canonical marker genes (EPCAM for epithelial cells, LYZ for myeloid cells, **Figure 1C**) classified cells into major types, including epithelial cells fibroblasts, and myeloid cells (**Figure 1A**). We observed significant differences in the cellular composition across tissues of distinct origins: normal, primary tumor, and metastatic lesion (**Figure 1B**). We then focused on the epithelial tumor cell population for subclustering analysis. This re-clustering identified several tumor subtypes with distinct functional states, such as a cycling phenotype (Tumor_Cycling), an epithelial-mesenchymal transition (EMT) phenotype (Tumor_EMT), and an inflammatory phenotype (Tumor_Inflammatory) (**Figure 1D**). Inference of copy number variation (CNV) confirmed that the Tumor_Inflammatory subpopulation exhibited the highest CNV score, indicating a higher degree of malignancy (**Figure 1E**). Further analysis of subpopulation dynamics (**Figures 1F, G**) revealed a progressive evolution during tumor progression: compared to normal tissue, the proportion of the inflammatory tumor subpopulation was enriched in primary tumors and further increased in metastatic lesions.

### 3.2 Single-cell profiling identifies and characterizes a pro-inflammatory malignant epithelial subpopulation enriched in metastasis

**Figure 2:**
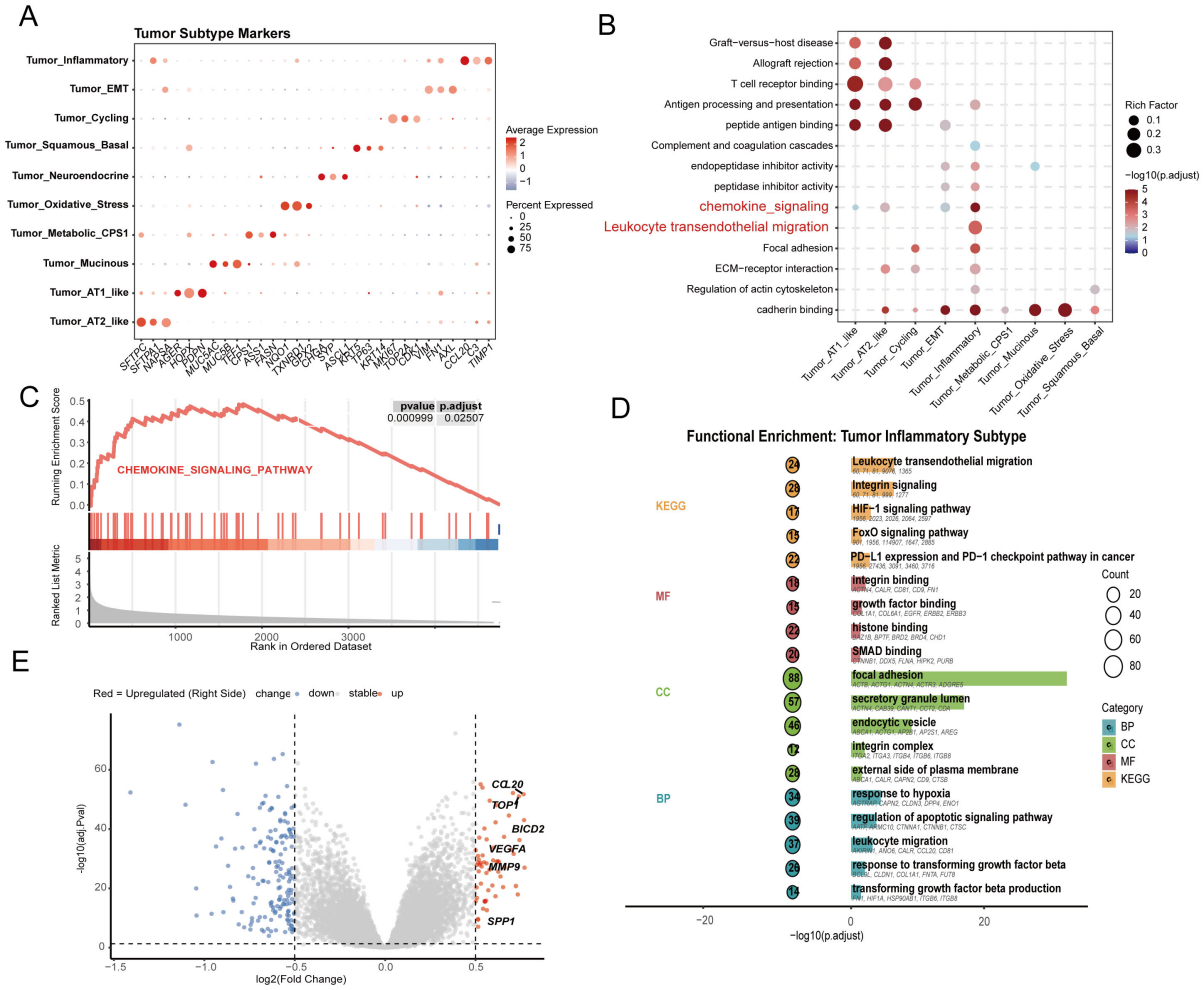
Marker genes, pathway enrichment, and key features of tumor subtypes, particularly the inflammatory subpopulation. **(A)** Bubble plot illustrating the expression patterns of subtype-specific marker genes across different tumor subpopulations. **(B)** Dot plot summarizing the results of Gene GO and KEGG pathway enrichment analyses for each tumor subcluster. **(C)** GSEA plot showing a significant enrichment of the “Chemokine signaling pathway” in the inflammatory tumor subpopulation. **(D)** Bar graphs depicting KEGG, Molecular Function (MF), Cellular Component (CC), and Biological Process (BP) enrichment analyses for the inflammatory tumor subpopulation, highlighting key immune-related pathways such as leukocyte transendothelial migration. **(E)** Volcano plot of differentially expressed genes between inflammatory and non-inflammatory tumor subpopulations, with CCL20 prominently labeled as a significantly upregulated gene.

To further characterize tumor cell heterogeneity at the single-cell level, we conducted an in-depth analysis of the identified tumor subpopulations. As illustrated in **Figure 2A**, we successfully identified specific marker genes for each tumor subtype. For instance, the Tumor_Inflammatory subpopulation exhibited high expression of inflammation-related genes such as MMP9 and CCL20, whereas the Tumor_EMT subpopulation was characterized by elevated expression of EMT-associated genes including AXL and TIMP1. Gene Ontology (GO) and Kyoto Encyclopedia of Genes and Genomes (KEGG) pathway enrichment analyses for each subpopulation (**Figure 2B**) revealed that the Tumor_Inflammatory subtype was significantly enriched for pathways related to “chemokine signaling” and “leukocyte transendothelial migration.”

To scrutinize the “inflammatory tumor subpopulation” in greater detail, we performed Gene Set Enrichment Analysis (GSEA), which confirmed the significant enrichment of the “chemokine signaling pathway” within this cluster (**Figure 2C**). Subsequent functional enrichment analysis (**Figure 2D**) highlighted that this inflammatory subpopulation was associated with a suite of pathways intimately linked to immune inflammation and tumor progression, including leukocyte transendothelial migration, integrin signaling, the HIF-1 signaling pathway, as well as PD-L1 expression and the PD-1 checkpoint pathway. Finally, differential gene expression analysis (**Figure 2E**) demonstrated that CCL20 was markedly upregulated in the Tumor_Inflammatory subpopulation and served as one of its key marker genes, further underscoring the potential role of CCL20 within the pro-tumor inflammatory microenvironment.

### 3.3 Single-cell trajectory and transcriptional regulatory analysis delineates the malignant evolution of LUAD and identifies a key regulatory axis

**Figure 3:**
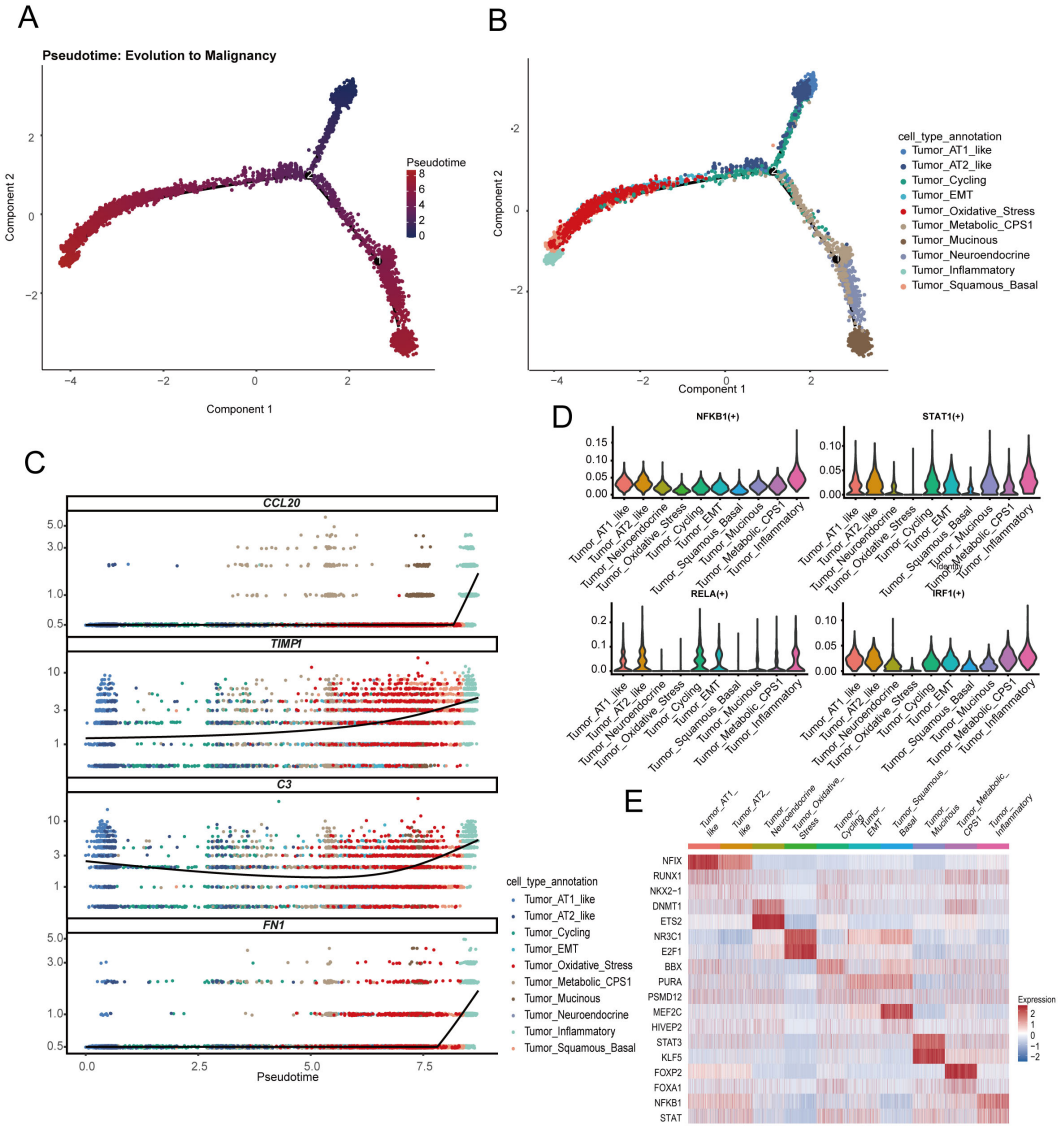
Trajectory inference and transcriptional regulatory network in tumor cell evolution. **(A)** Pseudotime trajectory of tumor cells constructed using the Monocle2 algorithm. The color gradient from dark blue to dark red represents the pseudotime progression, indicating the evolutionary direction from an early state toward a late, more malignant state. **(B)** The same pseudotime trajectory map annotated with tumor subpopulations. The results show that the Tumor_AT1/2_like subpopulation is predominantly located at the start of the trajectory, while the Tumor_Inflammatory and Tumor_EMT subpopulations are primarily distributed toward the trajectory end. **(C)** Expression dynamics of key genes (CCL20, TIMP1, C3, FN1) along the pseudotime trajectory. The fitted black curves illustrate the trend of gene expression over the evolutionary process, demonstrating a significant upregulation of CCL20 toward the late stage. **(D)** Violin plots showing the expression levels of key transcription factors (NFKB1, STAT1, RELA, IRF1) across tumor subpopulations, revealing their specific high expression in the Tumor_Inflammatory subpopulation. **(E)** Heatmap of regulon activity from SCENIC analysis. The columns represent individual cells, and rows represent transcription factors. The red color indicates high regulon activity, uncovering that NF-κB and STAT family transcription factors dominate the regulatory network in the inflammatory tumor subpopulation.

To delineate the malignant evolution of tumor cells in LUAD, we utilized the Monocle2 algorithm to construct a single-cell pseudotime trajectory (**Figure 3A**). This trajectory analysis revealed a continuous progression from a state resembling normal alveolar epithelial cells (Tumor_AT1/2_like, located at the trajectory origin) towards a dedifferentiated, malignant state (**Figure 3B**). Notably, the inflammatory tumor subpopulation (Tumor_Inflammatory) was predominantly enriched at the distal end of the trajectory, suggesting that this subpopulation represents a late-stage or highly malignant state in tumor evolution.

Analysis of gene expression dynamics along the pseudotime (**Figure 3C**) showed that CCL20 expression increased sharply as the trajectory progressed, peaking specifically in the inflammatory cells at the terminal phase of evolution. Concurrently, EMT-associated genes (TIMP1, FN1) were also upregulated along the trajectory, implying a cooperative role for inflammation and EMT in tumor progression.

To decipher the upstream regulatory mechanisms driving CCL20 overexpression and the formation of the inflammatory subpopulation, we reconstructed the transcriptional regulatory network using SCENIC analysis. Violin plots (**Figure 3D**) and regulon activity heatmaps (**Figure 3E**) showed that key transcription factors of the NF-κB signaling pathway (RELA, NFKB1) and interferon-response factors (STAT1, IRF1) exhibited high expression abundance and transcriptional regulatory activity within the inflammatory tumor subpopulation. Given that CCL20 is a canonical downstream target gene of the NF-κB pathway, these results suggest that during malignant evolution, tumor cells may activate the NF-κB/STAT signaling axis, thereby inducing CCL20 overexpression and shaping an immunosuppressive inflammatory microenvironment. It should be noted that the above analyses are computational inferences based on scRNA-seq data and currently lack direct experimental evidence (ChIP-seq or phosphoprotein detection) to confirm this regulatory pathway. Therefore, this upstream mechanism should be regarded as a hypothesis-generating exploration rather than a confirmed causal conclusion, warranting further functional validation.

### 3.4 Spatial transcriptomic analysis validates CCL20-mediated recruitment of tregs

**Figure 4:**
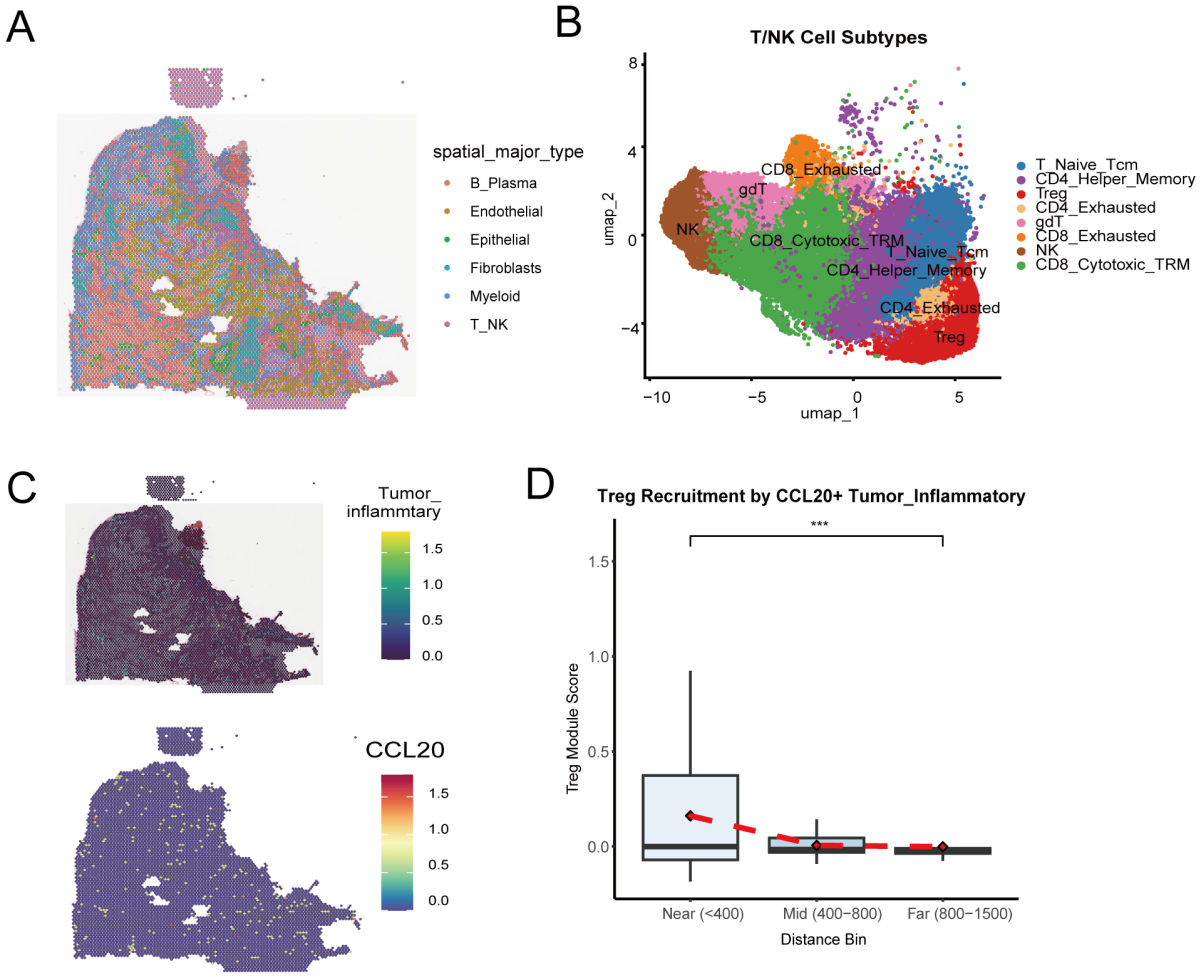
Spatial validation of the cellular architecture and the treg recruitment hypothesis. **(A)** Spatial transcriptomics atlas of a lung adenocarcinoma tissue section, depicting the spatial distribution of major cell types (epithelial, fibroblast, myeloid, T/NK cells). **(B)** UMAP re-clustering of T/NK cells, detailing lymphocyte subsets including Tregs and exhausted T cells. **(C)** Comparative spatial heatmaps of the inflammatory tumor signature score (top) and the Treg infiltration score (bottom). Brighter colors (yellow/green) indicate higher scores, visually demonstrating their substantial spatial overlap (co-localization). **(D)** Box plot of Treg enrichment analysis based on spatial distance. The x-axis represents distance intervals from the “inflammatory tumor region” (Near: <400 µm; Medium: 400-800µm; Far: 800-1500µm), and the y-axis shows the Treg module score. Results indicate a significant enrichment of Tregs in the near-distance region. ***, *P* < 0.001.

To validate the hypothesis of CCL20-mediated Treg recruitment within the authentic tissue architecture, we integrated spatial transcriptomic data. First, we constructed a spatial cellular atlas of the LUAD sample (**Figure 4A**), delineating the distribution boundaries between tumor cells and immune-stromal components. To precisely track Tregs, we performed sub-clustering and annotation of the T/NK cell population from our single-cell data, successfully identifying a definitive Treg subpopulation (**Figure 4B**). Subsequently, we mapped the previously defined gene signatures for the “Inflammatory Tumor Subpopulation (Tumor_Inflammatory)” and “Tregs” back onto the tissue sections (**Figure 4C**). Comparative analysis of their spatial heatmaps revealed a pronounced spatial co-localization: regions exhibiting high expression of the inflammatory tumor signature (bright areas in the upper panel) concurrently showed a specific increase in Treg infiltration abundance (bright areas in the lower panel).

To quantitatively corroborate this spatial proximity, we calculated the distribution density of Tregs across different distance intervals (**Figure 4D**). Statistical analysis demonstrated that within the immediate vicinity (<400 µm) of inflammatory tumor cells (Near region), the Treg module score was significantly higher than in mid-to-far distance regions. This key evidence spatially confirms that inflammatory tumor cells with high CCL20 expression form an immunosuppressive “hotspot,” capable of actively recruiting Tregs to their periphery, thereby fostering a microenvironment conducive to tumor survival.

### 3.5 Dissection of CCL20-CCR6-mediated intercellular communication underlying treg recruitment

**Figure 5:**
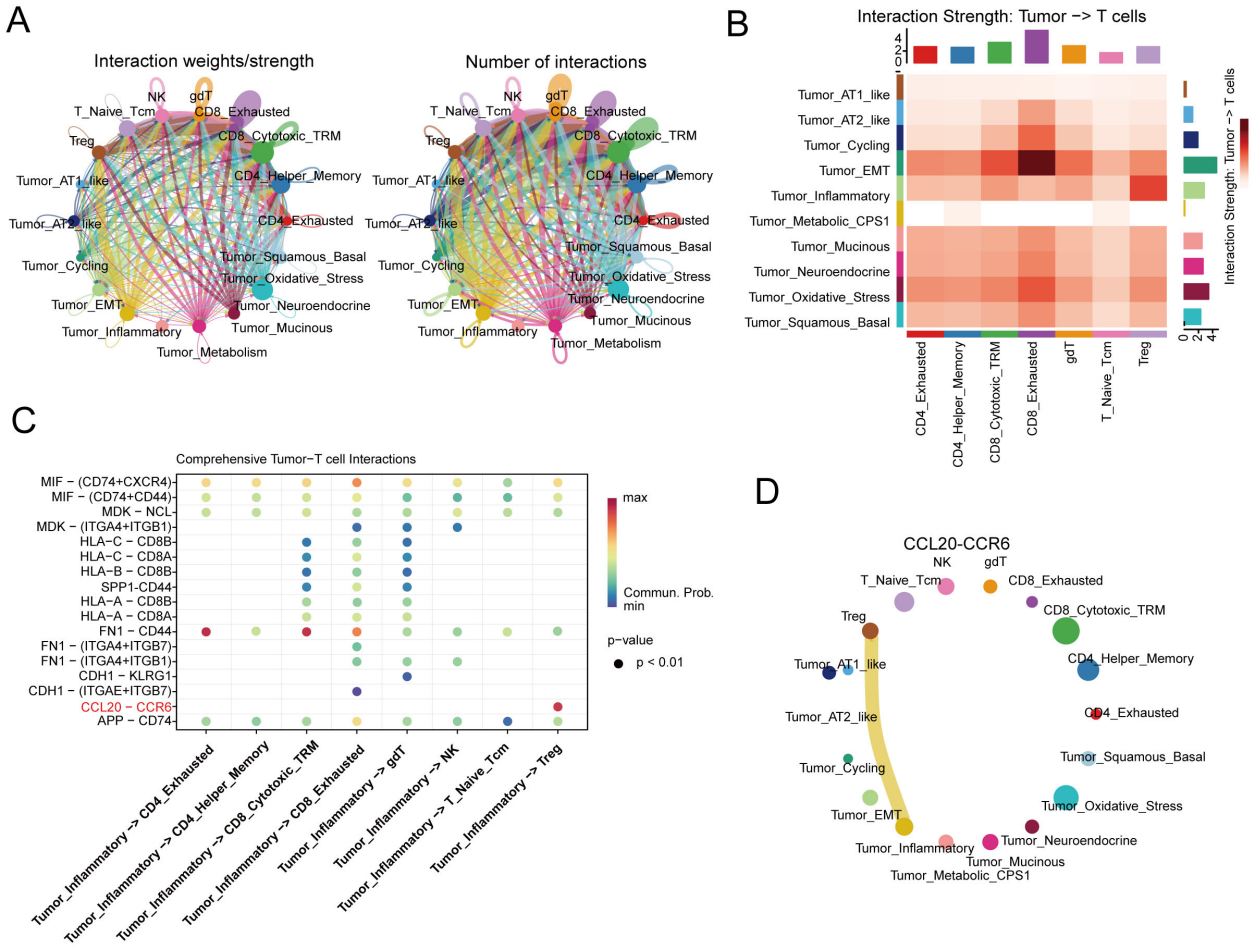
Cell-cell communication landscape between tumor subclusters and T cell subsets via cell chat analysis. **(A)** Cell communication network plots illustrating the overall interaction strength (left) and the number of interactions (right) between tumor subclusters and T cell subsets. Thicker and darker lines represent stronger communication. **(B)** Heatmap of interaction strength between different tumor subclusters as signal senders (Source) and T cell subsets as receivers (Target). Deeper red indicates stronger interaction, highlighting the potent signaling from the inflammatory tumor subpopulation (Tumor_Inflammatory) to Tregs. **(C)** Bubble plot of significant ligand-receptor pairs. This plot shows the molecular pairs significantly enriched in signals sent from the “Tumor_Inflammatory” subpopulation to various T cell subsets. Dot size represents the significance of the P-value, and color indicates the communication probability. The CCL20-CCR6 interaction targeting Tregs is highlighted in red. **(D)** Circos plot (circle plot) specifically depicting the CCL20-CCR6 signaling pathway. The arrow direction indicates signal flow, and the line width represents signal strength. The results clearly show that CCL20 signals are predominantly emitted by inflammatory and EMT-type tumor cells and specifically target Tregs.

To elucidate the molecular mechanisms underlying Treg recruitment, we systematically inferred the intercellular communication network within the TME using the CellChat algorithm. Global network analysis (**Figure 5A**) revealed extensive and intricate interactions between tumor cells and T cells. Notably, among all tumor subpopulations, the inflammatory (Tumor_Inflammatory) and EMT subpopulations exhibited the strongest outgoing signaling strength towards T cells (**Figure 5B**). This interaction intensity was particularly pronounced for communications with Tregs, significantly surpassing that of other, more benign or early-stage tumor subpopulations (Tumor_AT1/2_like). To pinpoint the key signaling molecules, we further analyzed the specific ligand-receptor pairs between the “Inflammatory Tumor Subpopulation” and various T cell subsets (**Figure 5C**). The bubble plot identified that, beyond common interactions such as MIF-CD74 and FN1-CD44, the CCL20-CCR6 ligand-receptor pair showed high significance and communication probability exclusively in the “Inflammatory Tumor Subpopulation → Treg” interaction. Finally, we extracted and visualized the CCL20-CCR6 signaling pathway from the complex network (**Figure 5D**). The chord diagram visually confirmed our central finding: the inflammatory tumor subpopulation serves as the primary source of CCL20 signals in the microenvironment, which specifically engage the CCR6 receptor on Tregs. This result molecularly completes the evidence chain of “malignant evolution/formation of inflammatory subpopulation/secretion of CCL20/recruitment of Tregs.”

### 3.6 Integrative multi-omics analysis identifies CCL20 as a hub gene and adverse prognostic biomarker in LUAD

**Figure 6:**
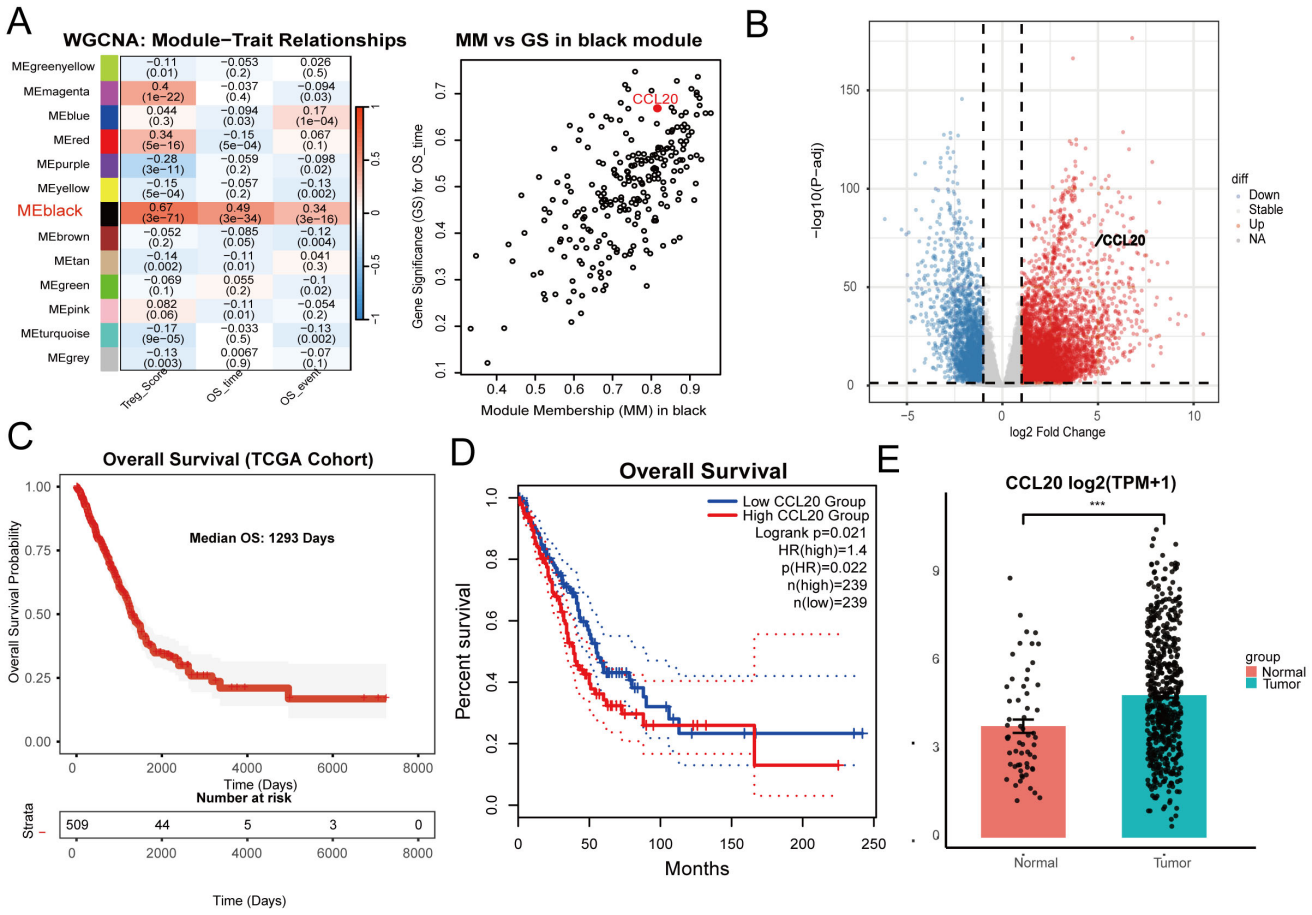
Expression profile and prognostic significance of CCL20 in the TCGA-LUAD cohort. **(A)** WGCNA Co-expression Network Analysis Identifies Key Modules and Hub Genes. Left: A heatmap illustrates the correlation between gene modules and clinical features (Treg score, survival status), revealing that the black module (MEblack) is highly correlated with Treg infiltration and survival events. Right: Scatter plot of module membership (MM) versus gene significance (GS) for genes within the MEblack module. CCL20 is identified as a top hub gene in this clinically relevant module. **(B)** Volcano Plot of Differentially Expressed Genes in TCGA-LUAD. The plot displays genes differentially expressed between lung adenocarcinoma tumor tissues and adjacent normal tissues. Upregulated and downregulated genes are shown in red and blue, respectively. CCL20 is highlighted as a significantly upregulated gene in tumors. **(C)** Baseline OS of the TCGA-LUAD Cohort. The Kaplan-Meier curve depicts the overall survival probability for the entire TCGA lung adenocarcinoma patient cohort included in this study. The median overall survival was 1293 days. **(D)** Kaplan-Meier Survival Analysis Based on CCL20 Expression Levels. Patients were stratified into high (cyan) and low (red) CCL20 expression groups. The CCL20 high-expression group showed a significantly worse overall survival rate compared to the low-expression group (*P* = 0.0041), indicating that high CCL20 expression is a marker of poor prognosis. **(E)** Quantitative Comparison of CCL20 Expression between Tumor and Normal Tissues. A boxplot with overlaid scatter points demonstrates that the expression level of CCL20 (log2 TPM + 1) is significantly elevated in lung adenocarcinoma tumor tissues (Tumor) compared to normal lung tissues (Normal). ***P < 0.001.

To identify key gene modules associated with Treg infiltration and clinical outcomes in LUAD, we performed a WGCNA. As shown in **Figure 6A**, the “black module (MEblack)” demonstrated a robust positive correlation with Treg scores and patient survival status. Subsequent analysis of genes within this module pinpointed CCL20 as a central hub gene. Differential expression analysis (**Figure 6B**) and paired-sample validation (**Figure 6E**) confirmed that CCL20 was significantly upregulated in tumor tissues compared to adjacent normal tissues. Subsequent survival analysis revealed (**Figures 6C, D**) that patients with high CCL20 expression exhibited a significantly shorter overall survival than those with low expression (HR = 1.41, P = 0.0041), suggesting that CCL20 serves as a potential adverse prognostic biomarker in LUAD.

### 3.7 Machine learning and multi-omics integration confirm CCL20 as a core biomarker and define its role in treg recruitment via CCR6 in LUAD

**Figure 7:**
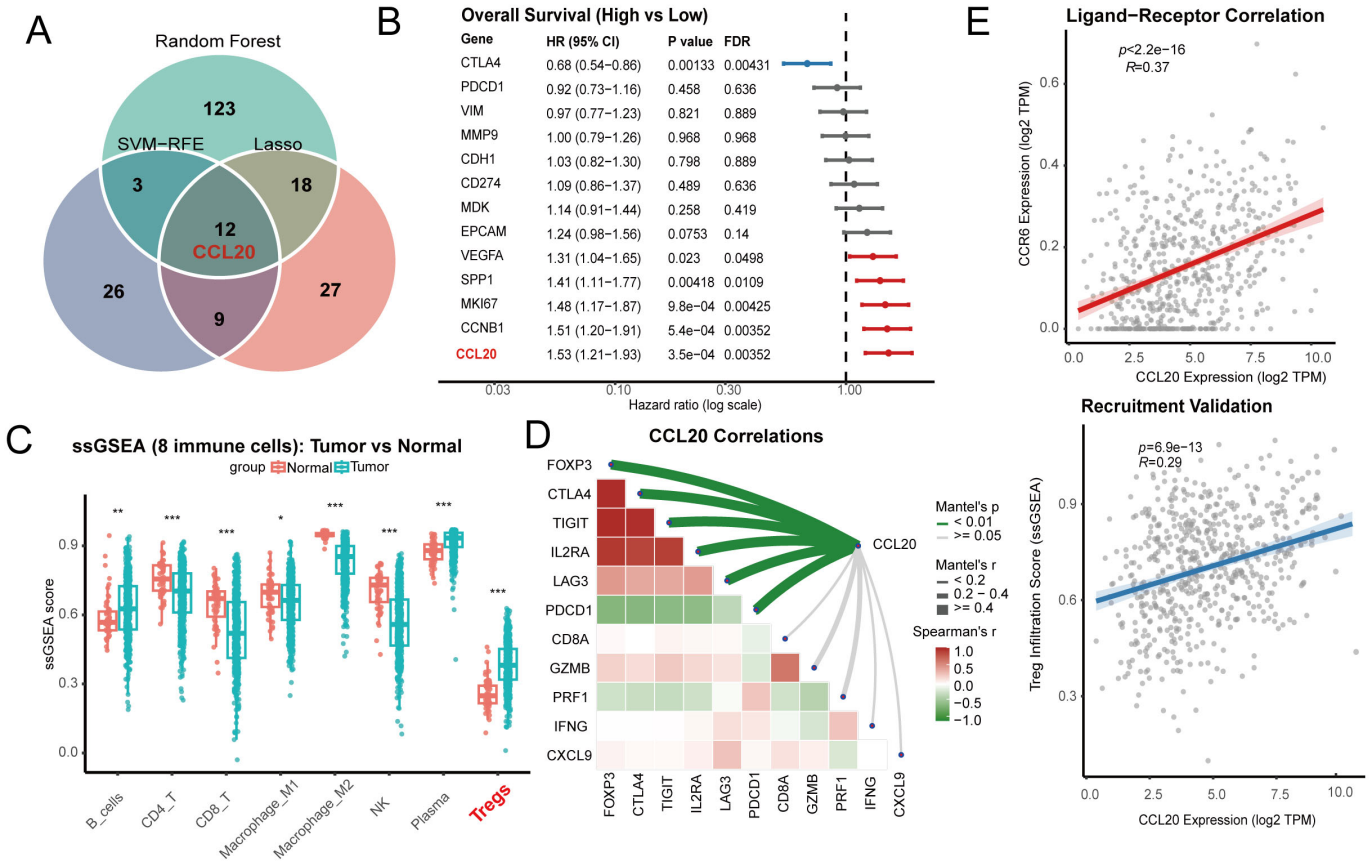
Validation of CCL20’s robust prognostic role and its mechanism in mediating treg recruitment into the LUAD. **(A)** Cross-Algorithm Feature Selection by Multiple Machine Learning Models. Three distinct algorithms (Random Forest, SVM-RFE, and Lasso regression) were applied for feature selection from the TCGA data. The Venn diagram shows that CCL20 is one of the 12 core genes identified by the intersection of all three methods, demonstrating its high robustness. **(B)** Univariate Cox Regression Analysis for Key Genes. The plot shows the hazard ratios (HRs) of multiple genes, confirming that high expression of CCL20 is a significant independent risk factor for patient death. This is contrasted with genes like CTLA4 and PDCD1. **(C)** Differential Immune Cell Infiltration in the Tumor Microenvironment (ssGSEA). The ssGSEA algorithm was used to assess the infiltration abundance of 8 immune cell types. Box plots demonstrate a significant increase in Treg infiltration scores in tumor tissue (Tumor) compared to normal tissue (Normal), indicating an immunosuppressive microenvironment. **(D)** Mantel Correlation Test between CCL20 and Treg-Specific Markers. The network plot on the right shows significant positive correlations (thick green lines) between CCL20 and the core Treg transcription factor FOXP3, as well as key surface molecules (CTLA4, TIGIT, IL2RA/CD25, LAG3), suggesting CCL20’s potential role in Treg maintenance or recruitment. **(E)** Validation of CCL20/CCR6 Co-expression and Treg Recruitment. Top: A correlation plot confirms a positive correlation between the ligand CCL20 and its exclusive receptor CCR6 at the mRNA level. Bottom: A correlation plot verifies the positive relationship between CCL20 expression levels and Treg infiltration scores (ssGSEA). * *P* < 0.05, ** *P* < 0.01, *** *P* < 0.001.

To further ensure the robustness of the biomarker, we integrated three machine learning algorithms—Random Forest, SVM-RFE, and Lasso regression—ultimately identifying 12 core candidate genes, including CCL20 (**Figure 7A**). This triple−algorithm consensus approach functions as an internal cross−validation strategy: only genes identified by all three methods—LASSO, SVM−RFE, and Random Forest—were retained, thereby reducing the risk of overfitting to any single algorithm or dataset and ensuring the robustness of the selected biomarkers. Univariate Cox regression analysis (**Figure 7B**) indicated that CCL20 conferred a hazard ratio (HR) of 1.53 (P<0.001), outperforming several traditional immune checkpoint genes. ssGSEA of the TME revealed a marked enrichment of Tregs in LUAD tissues (**Figure 7C**). For mechanistic exploration, correlation analyses (**Figure 7D**) demonstrated a strong positive correlation between CCL20 expression and both the Treg master transcription factor FOXP3 and surface inhibitory receptors (CTLA4, TIGIT). Furthermore, we confirmed a strong positive correlation at the mRNA level between CCL20 and its specific receptor, CCR6. Critically, CCL20 expression showed a significant positive correlation with Treg infiltration scores (**Figure 7E**), lending strong support to the hypothesis that CCL20 recruits Tregs into the TME via the CCL20-CCR6 axis to induce immunosuppression.

### 3.8 A Robust prognostic signature derived from the CCL20-CCR6 axis predicts high-risk patients in LUAD

**Figure 8:**
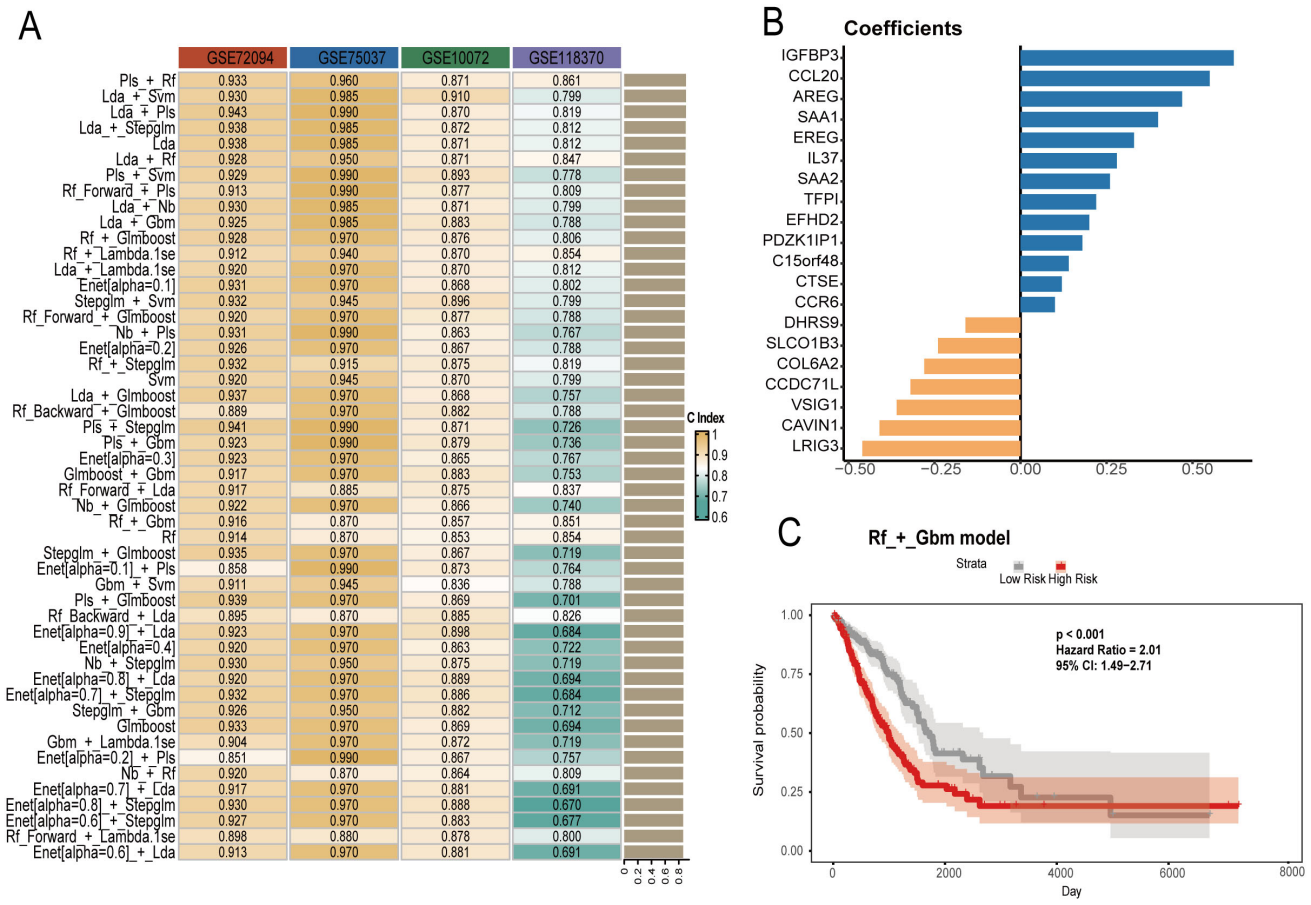
Development and external validation of a prognostic model Based on an ensemble of machine learning algorithms. **(A)** Heatmap of Algorithm Performance Screening. The heatmap displays the predictive performance (C-index) of dozens of algorithm combinations (Lasso, Random Forest, SVM, GBM) in the training set and multiple external validation cohorts (GSE75037, GSE10072, GSE72094, GSE118370). Darker colors (brown/yellow) indicate higher model prediction accuracy. **(B)** Feature Coefficients in the Optimal Model (Rf + Gbm). A bar chart shows the feature genes incorporated into the optimal model and their corresponding coefficients. Blue bars represent risk genes (coefficient > 0), and orange bars represent protective genes (coefficient < 0). Notably, CCL20 exhibits a high positive coefficient, identifying it as a primary risk-contributing factor. **(C)** Kaplan-Meier Survival Analysis Based on the Model-Derived Risk Score. The survival curve demonstrates that patients in the high-risk group have a significantly worse overall survival rate compared to those in the low-risk group (Hazard Ratio [HR] = 2.01, 95% Confidence Interval [CI]: 1.49-2.71, *P* < 0.001).

To translate the aforementioned biological insights into a clinically applicable prognostic tool and to validate the universal prognostic value of CCL20 across diverse cohorts, we conducted a comprehensive analysis integrating multiple external datasets, including GSE75037 and GSE10072. Employing an ensemble strategy, we systematically evaluated the predictive performance of various machine learning algorithms and their combinations, such as Random Forest (Rf), Support Vector Machine (Svm), Gradient Boosting Machine (Gbm), and Lasso regression. By comparing the concordance index (C-index), we found that the combined strategy of Rf + Gbm (feature selection by Random Forest followed by modeling with Gradient Boosting Machine) demonstrated the most stable and superior predictive capability across all validation cohorts (**Figure 8A**). Interrogating the internal feature weights of this optimal model (**Figure 8B**) revealed that CCL20 ranked as the second highest-weighted risk gene (Coefficient > 0), computationally reinforcing its role as a core molecular driver of poor prognosis in LUAD. Additionally, its receptor, CCR6, was also incorporated into the model as a risk factor.

Finally, stratifying patients based on the risk score calculated from this model (**Figure 8C**), survival analysis showed that patients in the high-risk group faced a 2.01-fold higher risk of death compared to those in the low-risk group. These results indicate that the prognostic model constructed based on CCL20 and related genes holds significant clinical potential for effectively identifying high-risk LUAD patients.

### 3.9 CCL20 knockdown suppresses oncogenic phenotypes in LUAD cells

**Figure 9:**
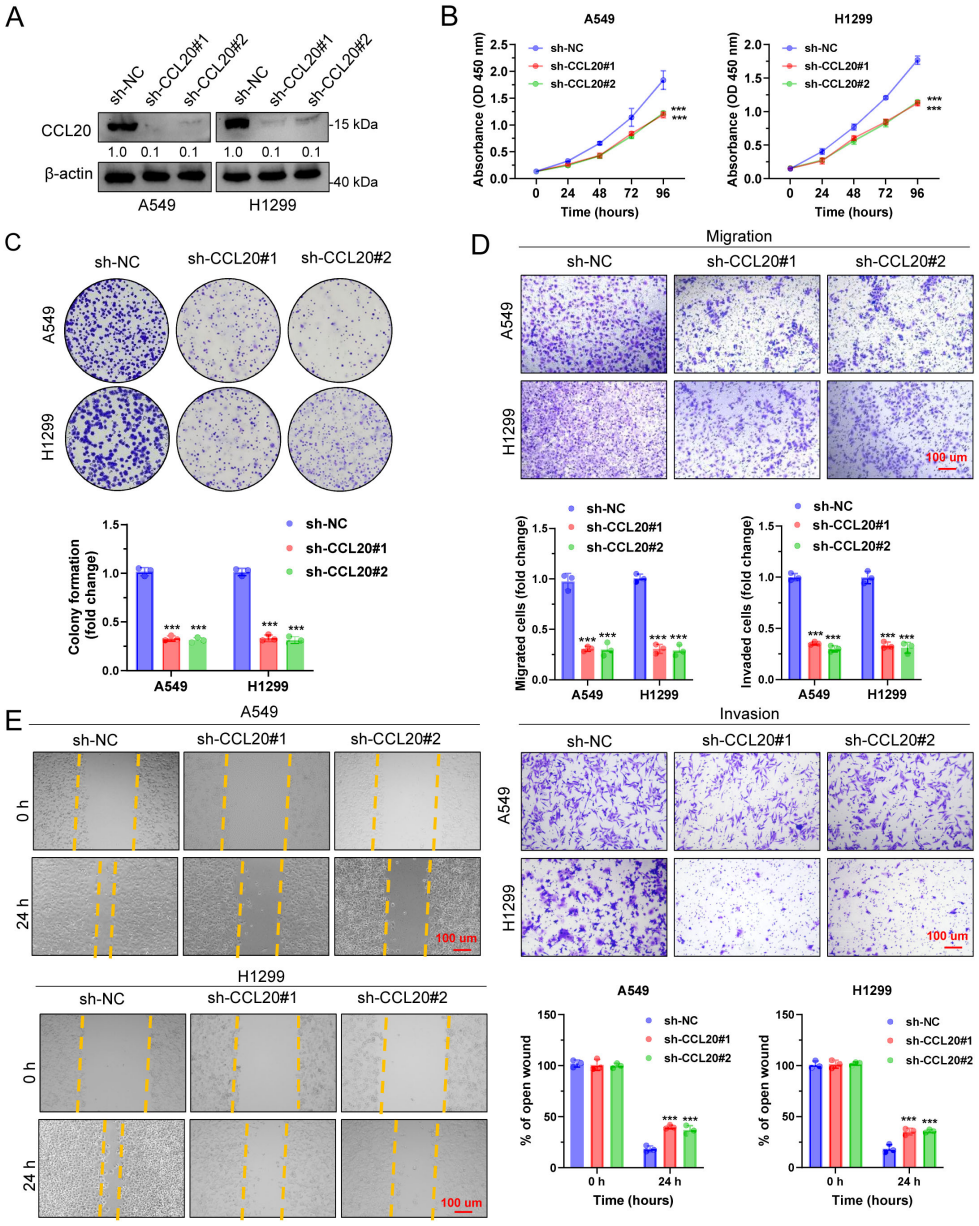
The oncogenic role of CCL20 in LUAD. **(A)** Western blot analysis confirms effective CCL20 depletion in A549 and H1299 cells following transduction with CCL20-targeting shRNA. **(B)** Proliferative capacity was assessed via CCK-8 assay at indicated time points post CCL20 knockdown. **(C)** A reduction in clonogenic potential is observed in cells upon CCL20 knockdown, as evidenced by colony formation assay. **(D)** The migratory and invasive abilities of cells were quantified using Transwell assays after CCL20 ablation. **(E)** Scratch wound-healing assay demonstrates a significant delay in gap closure upon CCL20 knockdown. Scale bars: 100 μm. Data are presented as mean ± SD from three independent experiments. *** *P* < 0.001. Statistical analysis was performed using a two-tailed Student’s t-test, as the data satisfied assumptions of normality and homogeneity of variance.

To functionally investigate the role of the elevated CCL20 observed in LUAD, we employed lentiviral-delivered shRNA to achieve stable knockdown of CCL20 in both A549 and H1299 cell lines. The successful reduction of CCL20 protein levels was verified via immunoblot analysis (**Figure 9A**). Assessment of cellular proliferation using a CCK−8 assay revealed a significant impairment in cell viability upon CCL20 silencing (**Figure 9B**). Furthermore, the long-term clonogenic potential was markedly diminished in cells depleted of CCL20, as shown by colony formation assays (**Figure 9C**).

We next evaluated the impact of CCL20 on cellular motility. Compared to control cells, the depletion of CCL20 resulted in a substantial decrease in both migratory and invasive capabilities, as quantified by Transwell assays (**Figure 9D**). Consistent with this, a scratch wound-healing assay demonstrated a pronounced delay in gap closure following CCL20 knockdown (**Figure 9E**). Collectively, these *in vitro* results demonstrate that targeting CCL20 curtails the proliferation, migration, and invasive properties of LUAD cells.

### 3.10 CCL20 depletion inhibits tumor growth *In Vivo*

**Figure 10:**
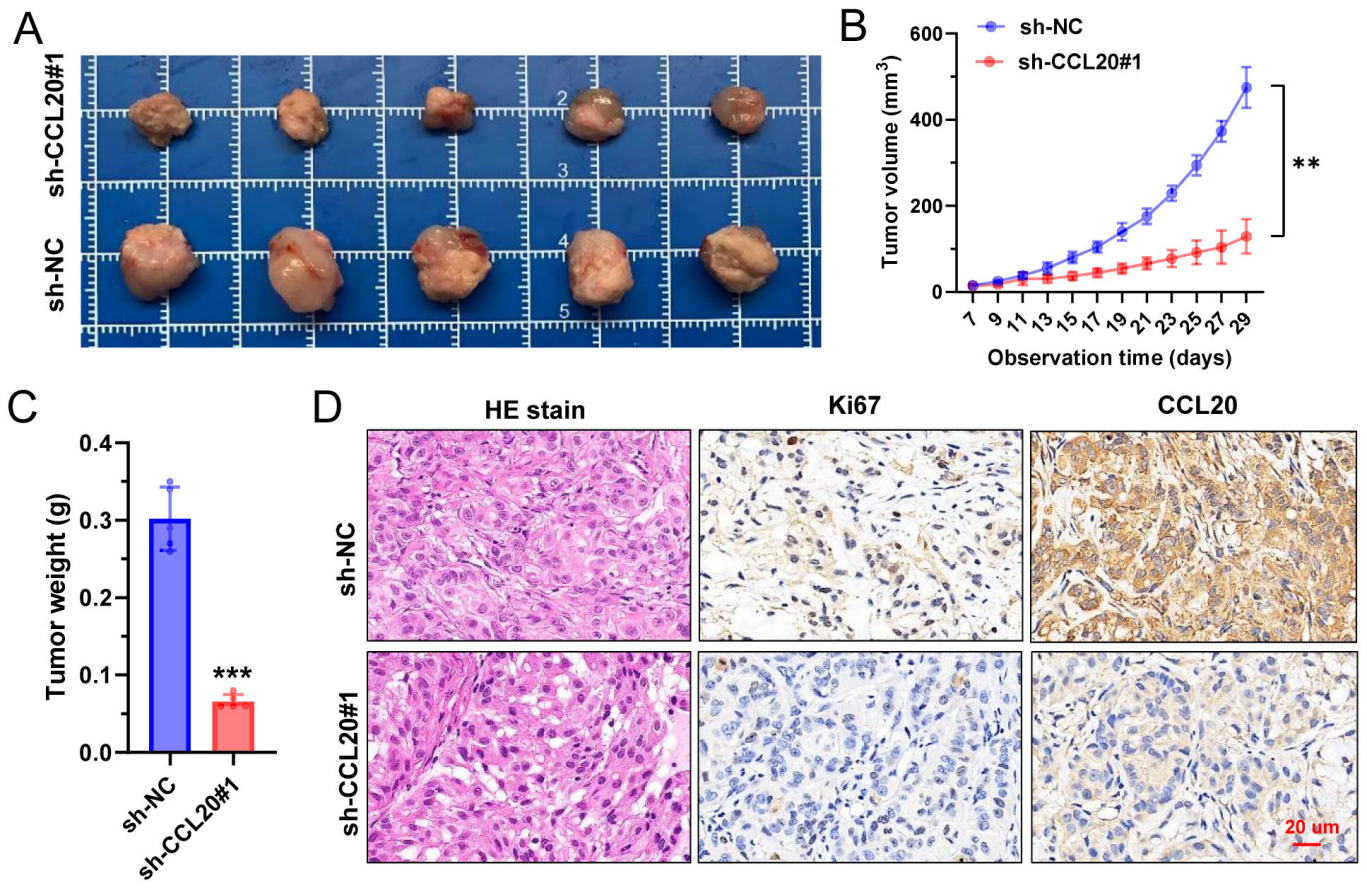
Attenuation of xenograft tumor growth *in vivo* following CCL20 ablation. **(A)** Representative macroscopic images of harvested tumor specimens from the indicated groups on day 29 post-implantation. **(B)** Tumor growth kinetics over time, monitored by serial volume measurements. **(C)** Bar graph depicting the final tumor weights assessed at the study endpoint (day 29). **(D)** Immunohistochemical analysis of tumor sections for the proliferation marker Ki−67 and CCL20 expression. Data are shown as mean ± SD (n=5 mice per group). ***P < 0.001, analyzed by two-tailed Student’s t-test. Scale bars: 20 μm. **P < 0.01, ***P < 0.001. Tumor volume and weight comparisons were analyzed using Student’s t-test.

To assess the functional role of CCL20 in tumor progression, we employed subcutaneous xenograft models. Over a 29-day observation period, mice implanted with A549 cells harboring sh-CCL20#1 demonstrated markedly attenuated tumor growth kinetics relative to the control group (sh-NC). This was evidenced by a slower increase in tumor volume and significantly decreased terminal tumor mass (**Figures 10A–C**). Immunohistochemical analysis of the resected tumors further revealed reduced levels of CCL20 alongside the proliferation marker Ki67 in the knockdown group (**Figure 10D**).

## 4 Discussion

Our study integrates single-cell and spatial multi-omics with functional validation to delineate a coherent mechanism by which a defined malignant epithelial subpopulation orchestrates an immunosuppressive microenvironment to fuel LUAD progression. We identify the chemokine CCL20 as a pivotal effector molecule in this process, linking tumor-intrinsic inflammatory signaling to the active recruitment of CCR6^+^ Tregs. These findings not only advance our understanding of LUAD biology but also highlight CCL20 as a candidate biomarker and therapeutic target.

The single-cell atlas revealed a metastasis-enriched, pro-inflammatory tumor subpopulation characterized by high genomic instability and CCL20 overexpression. This aligns with emerging concepts that intra-tumoral heterogeneity encompasses functionally specialized modules, with certain subpopulations acting as “niche engineers” (23, 24). Our trajectory and regulon analyses extend this view by demonstrating that this inflammatory state is not stochastic but emerges through a defined evolutionary path driven by the NF-κB/STAT signaling axis. NF-κB is a well-established master regulator of inflammation and cell survival in cancer, and its cooperation with STAT pathways can amplify pro-tumorigenic transcriptional programs (25). Our data directly link this core signaling nexus to the specific upregulation of CCL20, a known NF-κB target gene (26), thereby providing a mechanistic explanation for the origin of this chemokine-high state during malignancy.

A key contribution of our work is the spatial and functional validation of CCL20-mediated Treg recruitment. While prior studies have correlated CCL20 expression with poor prognosis or general immune cell infiltration in various cancers (27–31), the precise cellular source, target, and spatial logic remained inferential in LUAD. Our spatial transcriptomic data offer direct visual evidence that CCL20-expressing tumor cells and Tregs co-occupy specific tissue neighborhoods, creating immunosuppressive hotspots. This spatial context is crucial, as it suggests that CCL20 acts in a paracrine manner to establish localized, rather than diffuse, immune suppression. Thus, our work moves beyond prior studies that merely correlated CCL20 with prognosis or general immune cell abundance, by pinpointing the specific cellular source (inflammatory tumor subpopulation), target cell type (CCR6+ Tregs), and spatial architecture (localized immunosuppressive hotspots) that underpin its functional relevance. Furthermore, our CellChat analysis identified the CCL20-CCR6 interaction as the most highly ranked pathway between the inflammatory tumor subpopulation and Tregs based on its interaction score, in our dataset. This strengthens the biological plausibility of targeting this single ligand-receptor pair to disrupt the niche.

The translational relevance of the CCL20-CCR6 axis is strongly supported by our multi-omics analysis of bulk cohorts. CCL20 emerged as a central hub gene in a Treg-associated co-expression module and consistently demonstrated independent prognostic value across analytical methods. The robust correlation between CCL20 expression and established Treg markers (FOXP3, CTLA4, TIGIT) reinforces its role in shaping an immunosuppressive milieu. The prognostic model built using an ensemble machine-learning approach, which prioritized CCL20 and CCR6 as key features, successfully stratified patients across independent datasets. This moves beyond simple association, offering a potential clinical tool for risk assessment that is grounded in a concrete biological mechanism.

From a methodological perspective, our study illustrates the power of integrating multiple machine−learning algorithms for biomarker discovery. Rather than relying on a single statistical test or algorithm—which can be susceptible to dataset−specific biases—we employed a consensus pipeline that requires a candidate gene to be selected by LASSO, SVM−RFE, and Random Forest independently. This strategy dramatically increases the likelihood that the identified genes (CCL20) are genuinely associated with the outcome and not artifacts of a particular analytical method. Furthermore, the ensemble prognostic model (Rf + Gbm), which incorporates the top−ranked features from this consensus set, demonstrated stable and superior C−index across the training and multiple independent validation cohorts. This multi−layer, cross−validated framework offers a replicable template for future studies aiming to translate high−dimensional molecular data into clinically useful prognostic tools.

From a translational perspective, the integration of our CCL20-CCR6 axis-based risk score with established clinical staging systems (TNM) could provide added prognostic value. Currently, patients with the same TNM stage often exhibit heterogeneous outcomes, suggesting that molecular features may capture residual risk not accounted for by anatomical extent alone. By overlaying the continuous risk score onto TNM strata, clinicians could identify high-risk individuals within conventionally “low-risk” stages (Stage I or II) who might benefit from more aggressive surveillance or adjuvant therapy. Conversely, low-risk patients within advanced stages might be spared from overtreatment. Future studies should prospectively validate this integration using multivariable Cox models that include both the risk score and TNM components, and assess whether the combined model improves net reclassification improvement or decision curve analysis over TNM alone.

We acknowledge that our assignment of CCL20-CCR6 as the central pathway is based on a single computational tool (CellChat) and subsequent functional validation. This approach does not rigorously exclude the possibility that other chemokine-receptor axes contribute additively or synergistically. For instance, CXCL12-CXCR4, a well-known axis in cancer immune evasion, was detected in our dataset but did not rank as highly in the inflammatory subpopulation-Treg interaction. Nevertheless, direct competitive experiments—such as simultaneous blockade of multiple chemokine-receptor pairs (using neutralizing antibodies or small-molecule inhibitors) in a single *in vivo* model—would be necessary to establish the biological prioritized candidate pathway of the CCL20-CCR6 axis. Such studies represent an important direction for future work and could clarify whether CCL20-CCR6 is indeed the dominant driver of Treg recruitment in LUAD, or whether it acts in concert with other chemotactic signals.

Our functional experiments confirm the oncogenic role of CCL20 in LUAD. The impairment of proliferation, clonogenicity, migration, and invasion upon CCL20 knockdown *in vitro*, coupled with the significant suppression of tumor growth *in vivo*, establishes CCL20 as a bona fide tumor-promoting factor. The observed reduction in Ki67+ proliferating cells in CCL20-depleted tumors suggests that its role may extend beyond immune modulation to include direct or indirect effects on tumor cell fitness, possibly through autocrine signaling or by sustaining a permissive microenvironment. We acknowledge a critical disconnect between our proposed mechanism and the current *in vivo* evidence. The subcutaneous xenograft model in nude mice lacks functional T cells, including Tregs; therefore, the tumor growth inhibition observed upon CCL20 knockdown can be fully explained by the cell-autonomous effects of CCL20 on proliferation, migration, and invasion, as demonstrated *in vitro*. This model does not, and cannot, test the hypothesis that CCL20 promotes LUAD by recruiting Tregs. To directly evaluate the immune-mediated arm of our proposed model, future studies must employ immunocompetent models—such as a syngeneic mouse model (LLC1 or KP system) with intact T cells, or a transgenic model where the CCL20–CCR6 axis can be blocked specifically in Tregs. Only such models can resolve whether the recruitment of CCR6^+^ Tregs is indeed a necessary and sufficient mechanism through which CCL20 drives tumor progression *in vivo*.

Despite these insights, our study has limitations that point directly to specific future investigations. First, while spatial transcriptomics and CellChat analyses collectively support CCL20-mediated Treg recruitment, direct genetic proof of necessity and sufficiency—such as *in vivo* CCL20 overexpression in a CCR6 knockout background or pharmacological CCR6 blockade—remains the “gold standard.” Such experiments would definitively establish whether the CCL20-CCR6 axis is both necessary and sufficient for Treg accumulation in LUAD. Second, the therapeutic potential of this axis warrants exploration. Given that Tregs are a major barrier to effective anti-tumor immunity, disrupting their recruitment via CCR6 inhibition could synergize with immune checkpoint blockade (anti-PD-1 therapy). Preclinical models combining CCR6 antagonists with PD-1 inhibitors would test whether this combinatorial strategy can overcome Treg-mediated resistance and enhance anti-tumor responses. These two directions—mechanistic validation and therapeutic testing—represent a logical progression from our current findings toward clinical translation.

Looking forward, our findings open several therapeutic avenues. The CCL20-CCR6 axis represents a tangible target for disrupting a specific immunosuppressive pathway. Small-molecule inhibitors or monoclonal antibodies against CCR6 or CCL20 could be explored, particularly in combination with immune checkpoint blockade (anti-PD-1), to counteract Treg-mediated resistance (32). However, it must be emphasized that the realization of this translational potential depends on the validation of our core hypothesis in immunocompetent models using direct genetic approaches (such as CCR6 knockout or pharmacological blockade). Therefore, the most urgent future direction at this stage is not to initiate clinical trials directly, but to complete two “gold-standard” validations: first, to overexpress CCL20 in a CCR6 knockout background, or to pharmacologically block CCR6, to definitively establish whether this axis is necessary and sufficient for Treg accumulation; second, to evaluate the synergistic effect of CCR6 inhibitors combined with PD-1 inhibitors in syngeneic mouse models, to determine whether blocking Treg recruitment can enhance anti-tumor immunity. Only after these mechanistic insights are translated into experimental evidence can we advance the prognostic model based on this axis toward prospective clinical validation as a tool for identifying high-risk patients who might benefit from more aggressive or tailored adjuvant therapies.

In conclusion, our work unveils a detailed pathway wherein tumor cell evolution converges on CCL20 overexpression to architect an immunosuppressive niche via Treg recruitment. By bridging single-cell states, spatial ecology, multi-omics correlations, and functional validation, we have successfully elevated CCL20 from a correlative biomarker to a central mechanistic driver and a promising candidate for therapeutic intervention in LUAD. This complete chain of evidence not only answers the core questions of “where CCL20 comes from, where it acts, and how it works,” but also explicitly identifies the most critical gap in the current evidence (the lack of validation in immunocompetent models), providing a clear roadmap for subsequent mechanistic validation and therapeutic translation.

## 5 Conclusion

This study, through integrated multi-omics and functional analyses, delineates a key mechanism of immune microenvironment remodeling during LUAD progression. We demonstrate that enrichment of NF-κB/STAT signaling activity correlates with the emergence of a pro-inflammatory malignant epithelial subpopulation that highly expresses the chemokine CCL20. Via interaction with its specific receptor CCR6, CCL20 effectively recruits regulatory T cells (Tregs), thereby establishing a local immunosuppressive niche that facilitates disease progression. The primary causal evidence chain of our work is centered on the functional validation of the CCL20-CCR6 axis in Treg chemotaxis, which also forms the basis for a clinically relevant prognostic model. This study positions CCL20 as a potential key node linking tumor-intrinsic properties with immune microenvironment remodeling, a finding strengthened by cellular, spatial, and multi-omic evidence, and functionally supported. Thus, CCL20 represents a promising therapeutic target in lung adenocarcinoma.

## Data Availability

The original contributions presented in the study are included in the article/supplementary material. Further inquiries can be directed to the corresponding authors.
